# Evidence-based selection of training compounds for use in the mechanism-based integrated prediction of drug-induced liver injury in man

**DOI:** 10.1007/s00204-016-1845-1

**Published:** 2016-09-22

**Authors:** Sanja Dragovic, Nico P. E. Vermeulen, Helga H. Gerets, Philip G. Hewitt, Magnus Ingelman‐Sundberg, B. Kevin Park, Satu Juhila, Jan Snoeys, Richard J. Weaver

**Affiliations:** 1 AIMMS Section of Molecular Toxicology, Department of Chemistry and Pharmaceutical Sciences, VU University Amsterdam, De Boelelaan 1108, 1081 HZ Amsterdam, The Netherlands; 2 UCB BioPharma SPRL, Non-Clinical Development, Chemin du Foriest, 1420 Braine-l’Alleud, Belgium; 3 Early Non-Clinical Safety, Merck KGaA, Frankfurter Str. 250, 64293 Darmstadt, Germany; 4 Department of Physiology and Pharmacology, Section of Pharmacogenetics, Karolinska Institutet, 171 77 Stockholm, Sweden; 5 MRC Centre for Drug Safety Science, Department of Molecular and Clinical Pharmacology, University of Liverpool, Sherrington Building, Ashton Street, Liverpool, L69 3GE UK; 6 Orion Pharma, R&D In Vitro Biology, Orionintie 1A, P.O. Box 65, 02101 Espoo, Finland; 7 Pharmacokinetics, Dynamics and Metabolism, Janssen R&D, Turnhoutseweg 30, 2340 Beerse, Belgium; 8 Institut de Recherches Internationales Servier (I.R.I.S.), 50, rue Carnot, 92284 Suresnes Cedex, France

**Keywords:** Drug-induced liver injury (DILI), MIP-DILI, Set of training compounds, Evidence-based selection, DILI mechanisms

## Abstract

The current test systems employed by pharmaceutical industry are poorly predictive for drug-induced liver injury (DILI). The ‘MIP-DILI’ project addresses this situation by the development of innovative preclinical test systems which are both mechanism-based and of physiological, pharmacological and pathological relevance to DILI in humans. An iterative, tiered approach with respect to test compounds, test systems, bioanalysis and systems analysis is adopted to evaluate existing models and develop new models that can provide validated test systems with respect to the prediction of specific forms of DILI and further elucidation of mechanisms. An essential component of this effort is the choice of compound training set that will be used to inform refinement and/or development of new model systems that allow prediction based on knowledge of mechanisms, in a tiered fashion. In this review, we focus on the selection of MIP-DILI training compounds for mechanism-based evaluation of non-clinical prediction of DILI. The selected compounds address both hepatocellular and cholestatic DILI patterns in man, covering a broad range of pharmacologies and chemistries, and taking into account available data on potential DILI mechanisms (e.g. mitochondrial injury, reactive metabolites, biliary transport inhibition, and immune responses). Known mechanisms by which these compounds are believed to cause liver injury have been described, where many if not all drugs in this review appear to exhibit multiple toxicological mechanisms. Thus, the training compounds selection offered a valuable tool to profile DILI mechanisms and to interrogate existing and novel in vitro systems for the prediction of human DILI.

## Introduction

The appropriate selection of compounds for use in the evaluation of existing and novel model systems for the improved, and mechanism-based prediction of DILI in man requires special attention to overarching goals and strategies of the research programme. It is important to evaluate evidence for mechanisms that actually occur in patients. In the case of the MIP-DILI consortium (www.mip-dili.eu), the strategy of compound selection was to adopt a two-tiered approach towards compound selection. The first tier was to adopt an evidence-based evaluation of drugs with perceivably known mechanism of toxicity and propensity to cause liver toxicity in the human population. The first tier of compounds, defined as *training compounds*, would serve to probe existing and novel in vitro and in vivo model systems for use in defining a panel of improved assays for use in pharmaceutical research and development. The second tier, defined as *test compounds*, by contrast, comprises of legacy compounds from EFPIA Pharmaceutical companies previously known to cause liver injury in man or other (toxicology) species, but for which the mechanism(s) are poorly described. This paper describes the selection and compilation of known human DILI mechanisms on the set of training compounds identified for use in MIP-DILI.

**Fig. 1:**
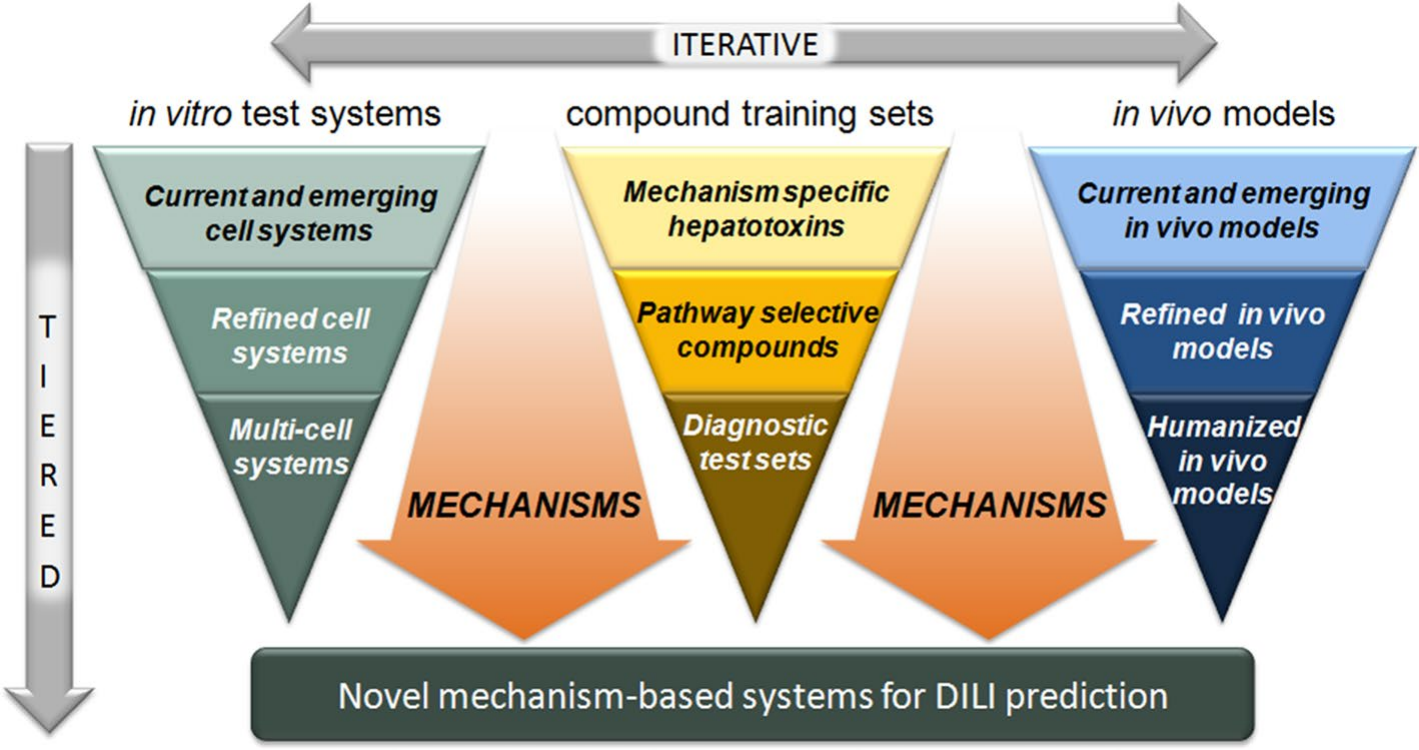
Schematic representation of the MIP-DILI project strategy

For the evidence-based training compounds, the MIP-DILI consortium aimed to use new knowledge gained during the project to further challenge existing and novel in vitro and in vivo systems for their value in both the understanding and prediction of DILI in man through complementary experimental and computational modelling paradigms to generate quantitative outputs for use in predictive drug liver injury through use of a tiered and iterative strategy (Fig. 1).

### Definition of training and test compounds

To support the concept of training and test compounds for use in the MIP-DILI consortium, definitions of each of these sets of compounds have been defined, as a consensus agreement, by which to select compounds for use in the evaluation model systems.

*Training compounds* were defined as suitable ‘Drugs associated with biological processes which are presently thought to be relevant to the initiation or prevention of DILI in man, alongside an appropriate negative control’.

*Test compounds* (EFPIA legacy compounds): ‘Drugs which are associated with DILI in either preclinical or clinical studies and could be potentially developed into a MIP-DILI training compound’.

### Mechanisms of drug-induced liver injury

The mechanisms by which most drugs cause liver injury are still poorly understood and subsequently hinder the detection of the hepatotoxic potential of drugs during pre-clinical and early clinical development. For the most part, mechanisms are based on studies in single in vitro or animal model test systems without any direct read across to the clinical situation. An essential step in the selection and use of training compounds and test systems is a consideration of their pharmacological and physiological relevance to man.

Many drugs are lipophilic substances and require transformation into metabolites by the phase I enzymes with subsequent conjugation by phase II enzymes to form water-soluble metabolites. While the majority of metabolites form readily excreted bio-inactive metabolites, phase I and to a lesser extent phase II enzymes also can metabolize drugs to electrophilic chemically reactive metabolites (CRMs) or unstable, reactive conjugates with the propensity to covalently bind macromolecular molecules in cells; the long-term persistent formation of which are believed to cause cellular dysfunction and liver injury (Srivastava et al. 2010). Furthermore, it is now clear that the intracellular concentration and persistence of a number of drugs is highly dependent on transporters which show variable expression. Knowledge of the disposition of training and test compounds in man is therefore vital for the interpretation of data from model systems. Coupled with the metabolic capacity of the liver, pre-existing liver disease, age, genetic variation, oxygen supply and the intrinsic properties of drugs, all are considered to predispose the liver to cellular injury and death.

Recent advances in our understanding of cell death modalities, based on biochemical and cellular insights, reveal a host of subtle yet distinct pathways defining programmed cell death through apoptosis and necrosis pathways, as opposed to the classical view of regulated and non-regulated cell death (Vandenabeele et al. 2010; Papatriantafyllou 2012). Recent research suggests necrosis, in part, is mediated through programmed cell death (necroptosis) through activation by tumour necrosis factor alpha (TNFα), Fas ligand (FasL) and tumour necrosis factor (TNF)-related apoptosis-inducing ligand (TRAIL) by ligands also implicated in apoptosis (Degterev et al. 2005). Necrotic cell death follows antioxidant consumption and oxidation of cellular macromolecular proteins, in turn potentially affecting mitochondrial membrane permeability, loss of mitochondrial membrane potential, decrease in ATP synthesis and disruption of Ca2^+^ homoeostasis. The mechanism of necroptosis is believed to be mediated through reactive oxygen species (ROS) production in response to TNFα activation (Lin et al. 2004).

In contrast to necroptosis, programmed cell death also occurs through the more widely established activation of a family of cysteine proteases and caspases, to mediate apoptotic cell death. Activation of caspases therefore occurs in response to agonistic death ligands, TNFα, FasL and TRIAL, and/or mitochondrial damage through the activation of pro-apoptotic BH-3 members of the BcL-2 family of ligands (Degterev et al. 2005; Christofferson and Yuan 2010). While drug-mediated necroptotic events are emerging for hepatotoxic drugs (Han et al. 2007; Dunai et al. 2012), it is widely accepted that many drugs cause liver injury through the caspase-dependent apoptotic pathways of cell death.

Apoptotic-inducing drugs are believed to alter the energetic state of mitochondria thereby placing the role of mitochondria as central to drug-mediated apoptotic hepatocellular injury and death. However, the previous classification of apoptotic and necroptotic pathways is perhaps now less clear for drug-related cell death, where roles for adenine nucleotide translocase and cyclophilin are implicated in membrane transition pore opening—a mechanism well recognized among several hepatotoxic drugs (Masubuchi et al. 2002, 2005).

Non-parenchymal cells may also be targets in DILI with the activation of the immune system and subsequent idiosyncratic reactions in which human leucocyte antigen (HLA) genotypes are implicated in the aetiology of these types of liver injury. Consideration of these mechanisms of DILI forms the basis for the selection of a training set of compounds based on clinical pathology of liver injury, but where the actual mechanism of DILI remains to be better defined. Therefore, *present day* knowledge of mechanisms of liver injury forms the basis for selection of training compounds that are relevant to liver injury at the cellular level. While drugs are central to the mechanism-based selection of training compounds involved in liver injury, non-drugs such as rotenone may be valuable as reagents for use in demonstrating the relevance of in vitro models for the prediction of DILI in man, particularly with respect to understanding specific molecular initiating events and molecular pathways leading to DILI. It must be noted that DILI can be a multi-step and multicellular event and therefore drugs may be ‘complex reagents’.

In addition to the selection of compounds with known mechanisms affecting mitochondrial function, are those known to cause liver toxicities as a consequence of changes in the physiological function of the bile acid transport network and steatosis. Among the many transporter proteins regulating the uptake and efflux of drugs and metabolites is the transport protein bile salt export pump (BSEP), the inhibition of which is implicated in the aetiology of several drug-induced liver toxicities.

The overarching goal of training compound selection is to develop a panel of best practice in vitro assays. However, the limitation of a mechanism-based strategy for the selection of training set of compounds relies on our present understanding of the mechanisms. The challenge, therefore, is to select training compounds based on our present mechanistic knowledge of cellular liver injury, yet contribute to our understanding and evaluation of existing and novel model systems for both the understanding and the prediction of liver injury in man.

### Acute and chronic forms of liver injury

The rationale of chronic versus acute dosing is to delineate if acute dosing and repeated dosing leads to the same mechanism of toxicity (Ramachandran and Kakar 2009). For example, acute toxicity of CCl4 leads to centrilobular necrosis, but on cessation the liver fully recovers. By contrast, long-term exposure or repeated exposure leads to a centrilobular adaptation and finally to peripheral fibrosis. Overdose of APAP by contrast leads to necrosis with either fulminant liver failure or recovery and no fibrosis even following long-term repeated administration. Nitrofurantoin is another example that, on the one hand, can cause rare reversible acute liver injury and, on the other hand, more common chronic hepatitis with autoimmune-like features.

These examples of liver toxicants illustrate the a priori requirement to select training compounds on the basis of an understanding of the mechanism(s) of liver injury, such that these mechanisms are recapitulated in in vitro cellular-based assays. Nevertheless, in the selection of training compounds, the challenge is to understand these mechanisms as they relate to in vivo liver injury in human in both acute and chronic drug administration in patient populations.

### Compound selection criteria

Training compound selection was made by representatives from both EFPIA companies and academia in the MIP-DILI consortium using data compiled by partners and assembled for evaluation in a purposed built Central data repository (CDR) for the compilation of background and foreground data. The goal was to select 10 training compounds positive for their propensity to cause DILI in man and, where possible, chemically matched negative compounds.

**Fig. 2:**
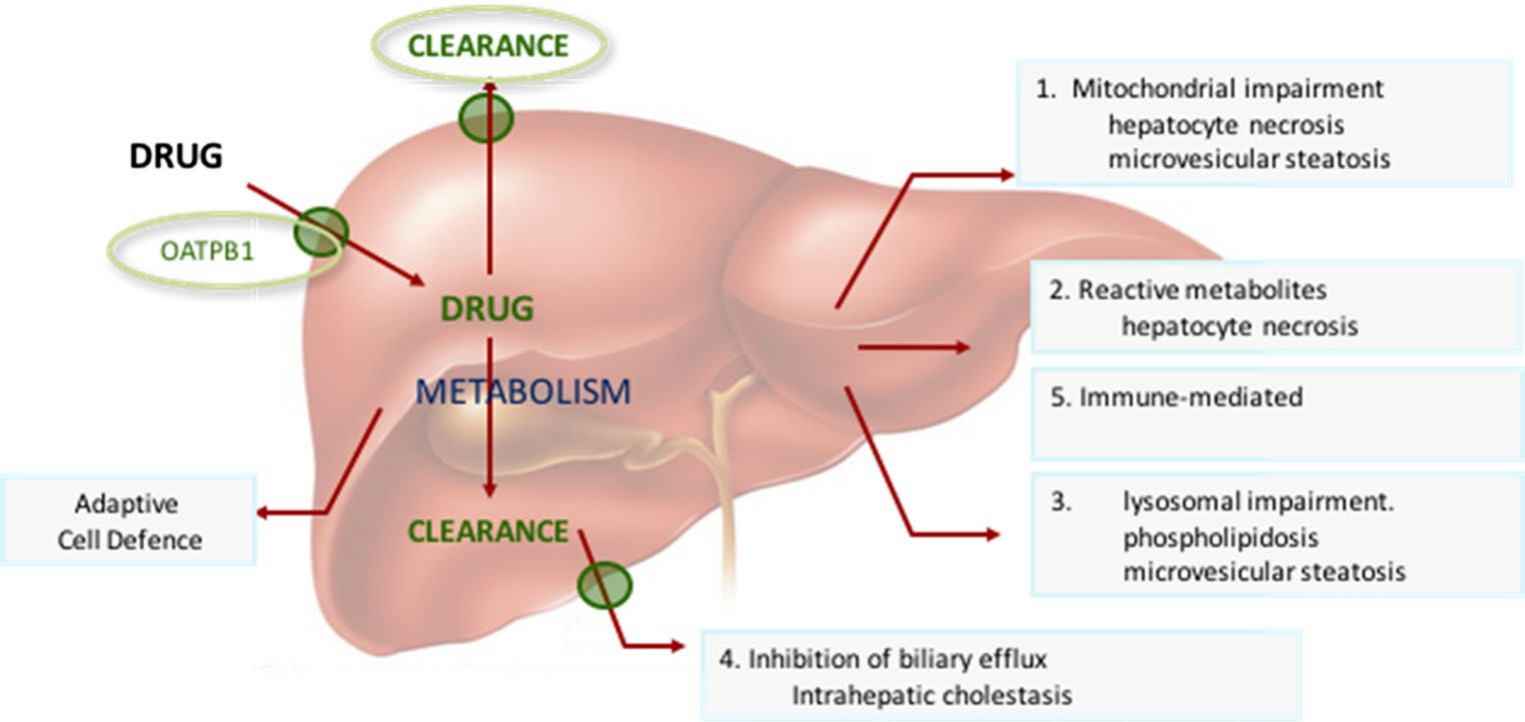
Illustration of MIP-DILI relevant mechanisms of drug induced liver injury in man

Criteria for the selection of training compound are summarized in Table 1. The selection of training compounds was identified to broadly cover liver toxicity as defined by the perceived or known mechanisms of chemical insult and hepatocellular injury. The mechanisms of hepatocellular injury are broadly classified into five main areas namely (1) mitochondrial dysfunction, (2) chemically reactive metabolites and necrotic toxicity, (3) lysosomal dysfunction, phospholipidosis and steatosis, and (4) bile transport inhibition for the study of mechanisms of cellular injury in in vitro cellular-based models, and to include (5) adaptive or immune-mediated mechanisms believed to involve, but not restricted to, HLA phenotypes (Fig. 2).

**Table 1:** Criteria for evidence-based selection of training compounds for the MIP-DILi project

DILI category	Model hepatotoxins that selectively target specific pathways/systems in the hepatocyte
	Model hepatotoxins that cause specific forms of DILI in preclinical model systems
	Drugs that have a well-defined association (clinical phenotype, frequency, severity) with particular forms of DILI in man and in non-clinical models
	Drugs that cause DILI in man but did not in available non-clinical test systems
	Compounds that do not show liver damage either in pre-clinical tests or in man, but which are chemically related to drugs that are clearly associated with DILI, to act as negative controls
Mechanism known	Molecular target
	Reactive metabolites
	CYP independent cell injury
	Mitochondrial impairment
	Inhibition of BSEP
	Innate/adaptive immune activation
	Other
DILI initiating primary event	Evidence for primary event (in vitro/in vivo)
	Evidence for mechanism (in vitro/in vivo)
	Drug or metabolite involved
	Dose–response
ADME data available?	Characterization of drug exposure and metabolite profiles (Phase I–III)
DILI frequency in humans?	Clinical evidence of liver injures reported
Human-specific DILI?	Evidence of human only mechanism(s) of liability and liver injury
Human selective DILI?	Sensitivity as it relates to in vivo or in vitro test systems

Compound selection focused on marketed or formally marketed drugs known to exhibit DILI in man, including both dose-dependent and idiosyncratic and/or dose-dependent toxicities. Of the 40 compounds nominated, 10 compounds were selected as training compounds for use in the initial phase of the research programme (Table 2). Acetaminophen (APAP), amiodarone, diclofenac, fialuridine and tolcapone were selected on evidence of mitochondrial dysfunction; APAP, diclofenac, nefazodone, tolcapone, and troglitazone on the formation of reactive metabolites; amiodarone, perhexiline, and troglitazone on causing lysosomal impairment and bosentan, diclofenac, nefazodone, perhexiline, and troglitazone as inhibitors of transport proteins (Table 2). Ximelagatran and flucloxacillin were selected among a number of immune-mediated liver toxicities. Where possible, matched negative controls were identified, which in the case of tolcapone and troglitazone were entacapone and pioglitazone, respectively. Chemical structures of MIP-DILI training compounds and negative controls are shown in Table 3.

**Table 2:**
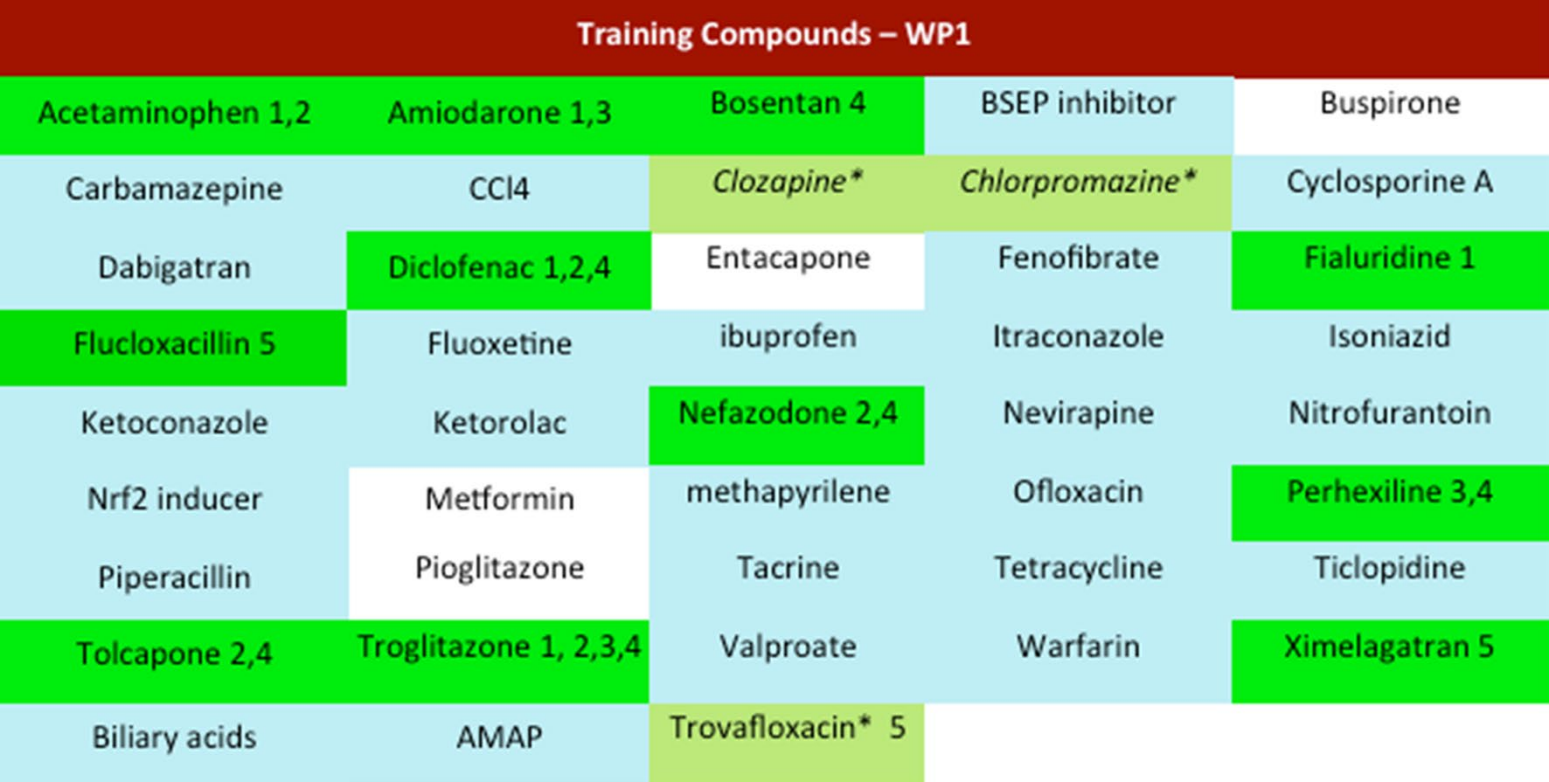
Panel of potential training compounds and the ultimate selected training compounds (green), four negative controls (white) and still to be decided (light green)

Mechanisms: *1* mitochondrial, *2* reactive metabolites, *3* lysosomal impairment, *4* BSEP inhibition, *5* immune-mediated

**Table 3:**
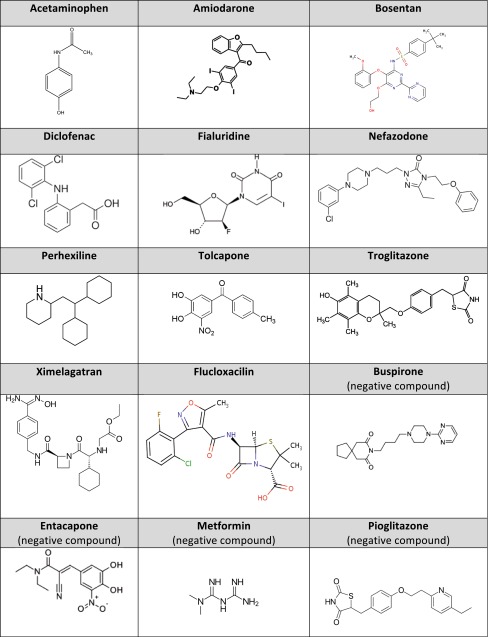
Chemical structures of MIP-DILI training compounds

## Idiosyncratic toxicity and toxicity profiles of selected training compounds

### Idiosyncratic toxicity

Drug-induced liver injury can be predictable and unpredictable (‘idiosyncratic’). Predictable liver toxicity is dose dependent and, essentially, all patients will develop liver injury if they receive a sufficiently high dose of the hepatotoxic drug. The toxicity of APAP remains one of the most extensively studied drugs known to cause dose-dependent liver toxicity in humans and preclinical species (Larson 2007). The term idiosyncratic means that the occurrence of DILI is a function of the individual. Such reactions may have an immunological basis, but this must not be assumed.

Alternatively, drugs known to form chemically reactive metabolites (CRMs) can be associated with idiosyncratic toxicity. These drugs which form CRMs provide evidence of a possible link between reactive metabolite formation and idiosyncratic liver toxicity—not necessarily dose-dependent, occurring only in a small fraction of susceptible individuals and within the intended therapeutic range (Uetrecht 2000). Idiosyncratic adverse drug reactions (IDRs) associated with liver injury are reported in 50 % of the cases of acute liver failure (Abboud and Kaplowitz 2007), but the frequency of IDRs is low accounting for 1 in 10,000–1 in 100,000 patients. IDRs are often not identified until the drug reaches a large patient population. In Table 4, an overview is given of the selected training compounds involving the relevant processes and mechanisms in DILI.

**Table 4:** Overview of relevant processes and mechanism involved in DILI of training compounds

Training compound	1	2	3	4	5
	Mitotoxicity	CRM	Lysosomal	Biliary	Immune
Fialuridine	✓				
Acetaminophen	✓	✓			✓ (Innate)
Diclofenac	✓	✓		✓	
Amiodarone	✓		✓		
Perhexiline			✓	✓	
Bosentan				✓	
Tolcapone	✓	✓		✓	
Nefazodone		✓		✓	
Troglitazone	✓	✓	✓	✓	
Ximelagatran					✓ (Adaptive)
Flucloxacillin					✓ (Adaptive)

Below the relevant mechanisms for DILI of the training compounds will be illustrated using representative training compounds. For details on the relevant processes and mechanisms involved in DILI of all training compound, reference is made to Table 5.

**Table 5:** Details of relevant processes and mechanism involved in DILI of the training compounds

Training compound	Phase I	Phase II	Phase III and BSEP inhibition	Cell death	Mitochondrial dysfunction	Lysosomal dysfunction and phospholipidosis	Immune response	Association studies
Acetaminophen	NAPQI, CRM responsible for APAP-hepatotoxicity CYP2E1 is primary enzyme, other CYPs (including CYP1A2 and CYP3A4) also involved in bioactivation At therapeutic dose NAPQI is efficiently deactivated through spontaneous or enzymatic (GST) GSH conjugation Overdose of APAP results in GSH depletion and protein binding	Mainly glucuronidation and sulfation APAP-glucuronide (UGT1A1 and 1A6): 50–70 % of the dose APAP-sulphate (SULT1A1, 1A3/4 and 1E1) accounts for 25–35 % of the dose At a therapeutic dose 5—15 % excreted in urine as mercapturic acid or cysteine conjugate	Unlike APAP, elimination of APAP metabolites require transporters Biliary excretion: APAP-glucuronide—MRP2 and BCRP, APAP-sulphate: MRP2 and BCRP, APAP-GSH: MRP2 Basolateral excretion: APAP-glucuronide: MRP3, APAP-sulphate: MRP3 and MRP4	Majority of animal studies suggest necrosis Increasing number of reports suggest apoptosis also plays a key role	Associated with binding of NAPQI to mitochondrial proteins			
Amiodarone	Primarily metabolized to its active metabolite, mono-*N*-desethylamiodarone (DEA) by several hepatic CYPs (primarily by **CYP3A4** but also **CYP1A1, 2D6** and **2C8**) Both, Amiodarone and its major active metabolite contribute to observed overall hepatotoxicity	Glucuronide conjugation is an important elimination pathway of amiodarone and its phase 1 metabolites (conjugates found in human bile)	Amiodarone and DEA are excreted through the biliary system Inhibitory effects of amiodarone towards Phase 3 transporters observed (primarily uptake, OATP2) MDR1 possibly plays a role in elimination of amiodarone (induced in rat) Mdr2 expression progressively decreases upon amiodarone treatment	Micro- and macrovesicular steatosis, phospholipid laden lysosomes (phospholipidosis, impairment of lysosomal function), intense ballooning of hepatocytes, presence of abundant mallory bodies, and fibrosis Pseudoalcoholic hepatitis End-stage liver disease occurs rarely in human probably due to repair	Inhibition of mitochondrial beta-oxidation → triglyceride accumulation and microvesicular steatosis Interference with mitochondrial energy production, disruption of electron transport chain enzyme complexes and uncoupling of oxidative phosphorylation subsequent to amiodarone accumulation in mitochondria Severe mitochondrial impairment → ATP depletion and subsequent cell death	Accumulation in lysosomes and trapping due to protonation in acidic environment Amiodarone complexes with phospholipids and inhibits intralysosomal phospholipase A resulting in accumulation of phospholipids		
Bosentan	Metabolites are reported to inhibit rat BSEP Reactive metabolites +are formed, but not thought to play a role through the formation of ROS **CYP3A4** and **CYP2C9** dependent metabolism	UGT1A1 dependent metabolism and transport	Prototypical inhibitor of BSEP dependent transport Substrate (MRP2) and stimulates MRP2 activity, uncoupling bile dependent (BSEP) and independent (MRP2) flow inducing cholestasis Trans inhibition of BSEP ruled out					
Diclofenac	Oxidation of DF at 4′ position by CYP2C9 major oxidative route Minor route is 5-OH-DF formation by CYP3A4 Both 4′OH-DF and 5OH-DF form reactive quinone imines, inactivated by GSH Quinone imines are protein reactive and implicated in redox cycling/causing oxidative stress Minor bioactivation pathways result in reactive arene oxide (CYP2C9) and *o*-imine methide (CYP3A4), inactivated by GSH Multiple minor oxidative routes result in stable metabolites NQO1 and NQO2 reduce QIs back to the OH– metabolites	Glucuronidation is major route → diclofenac acyl-glucuronide (DF–AC), mainly by UGT2B7 but also by UGT1A9 and 1A6 Subsequent hydroxylation of DF–AC by CYP2C8 results in 4-hydroxy acyl-glucuronide formation DF–AC is protein and GSH reactive GSH conjugation results in a reactive DF-*S*-acyl-glutathione thioester Quinone imines inactivated by GSH S-transferase (GST) catalysed GSH conjugation	DF is efficiently transported by human Bcrp1 and to a lesser extent by BCRP DF–AC, hydroxy acyl-glucuronides as well as 4′hydroxy and 5-hydroxy diclofenac are excreted into bile by ABCC2 In addition Bcrp1 and Mrp3 are also involved in the biliary excretion and sinusoidal efflux of DF–AC, respectively	At low concentrations apoptosis by mechanisms related to MPT induction When a large proportion of the mitochondria are affected, necrotic cell death can occur Metabolism related cell death in rat hepatocytes → inhibition of CYP activity reduced cell toxicity while inhibition of UGT aggravated the toxicity	DF induces mitochondrial permeability transition (MPT) 4′hydroxy DF, DF–AC and DF glutathione thioester are stronger inhibitors of ATP synthesis compared to DF No mitochondrial toxicity observed in HepG2 gal-glu assay at low concentrations		Immune cell activation by 5-hydroxy-DF and DF-2,5-quinoneimine in mice; DF and DF-AC no immune cell activation Hepatic adducts and circulating antibodies in patients with hepatotoxicity	Association with UGT2B7*2, CYP2C8, and ABCC2 polymorphisms: increased formation of reactive DF metabolites GWAS study: association UGT2B7; no HLA association Association GSTM1 and T1-null genotype
Flucloxacillin	Hydroxylation of the 5-methyl group in the isoxazole ring to a 5-hydroxymethyl derivative by **CYP 3A4**		An inhibitor of BSEP				Activates CD4+ and CD8+ T cells in patients with DILI Drug-specific CD8+ T cell response HLA-B*57:01 restricted	GWAS study showed HLA-B*57:01 as major determinant of DILI
Fialuridine	FIAU to FMAU to FAU (via thymidylate synthase)—FMAU also has activity against mtDNA following phosphorylation	Unknown glucuronide conjugates reported Phosphorylation through mono-phosphate (thymidine kinase), di-phosphate (thymidylate kinase) to tri-phosphate (DPK) species	Localized to mitochondria via hENT1	Inhibition of mitochondrial DNA polymerase γ by the triphosphate metabolite → depletion of mtDNA Loss of mtDNA encoded proteins leads to loss of function of mitochondrial respiratory chain				
Nefazodone	Metabolized to active hydroxynefazodone, m-chlorophenyl-piperazine and triazoledione Inhibitor of CYP3A4 in vitro and in vivo Extensively metabolized in the liver by n-dealkylation, aliphatic and aromatic hydroxylation less than 1 % of intact drug recovered in urine The clearance of mCPP is CYP2D6 dependent High first-pass metabolism and plasma protein binding	Metabolites are finally eliminated as glucuronide or sulphate conjugates	Strong inhibition of BSEP Inhibits the efflux activity of MDR1	Apoptotic cell death	Mitochondrial dysfunction and subsequent apoptotic HepG2 cell death in addition to marked cytosolic calcium increase Inhibition of mitochondrial respiratory complex I and IV, associated with accelerated glycolysis → mitochondrial membrane potential collapse, GSH depletion and oxidative stress			
Perhexiline	Hydroxylation by CYP2D6 (highly polymorphic, 10–20-fold inter-individual variability)	Secondary metabolism to dihydroxy-metabolites and glucuronide conjugates	No reports of interaction		Protonated perhexiline accumulates in mitochondrial membrane, leading to multitude of effects, incl. uncoupling of mitochondrial oxidative phosphorylation, inhibition of complexes I and II, decreased ATP formation and reduced mitochondrial β-oxidation of long-chain fatty acids → triglyceride accumulation and microvesicular steatosis and/or cell death	Non-covalent complexes of protonated perhexiline with phospholipids are formed which inhibit the action of intralysosomal phospholipases resulting in accumulation of phospholipids along with lysosomal accumulation of the drug		
Tolcapone	Several metabolites derived from reduced drug at 5-nitro Oxidation of amine and acetylamine by several P450 s (CYP2E1, −1A2) to reactive species that could be trapped with glutathione (GSH) Oxidative hydroxylation of the methyl group by CYP3A4 and to the carboxylic acid is a minor pathway CYP2C9 as major P450 in hydroxylation of tolcapone	The major early and most abundant metabolite is 3-*O*-β,d-glucuronide conjugate; UGT polymorphism The major late metabolite in plasma is 3-*O*-methyltolcapone (methylation by COMT) *N*-acetylamino glucuronide in urine and faeces	Modest inhibition of MRP3, MRP4 and BSEP		Uncoupling of oxidative phosphorylation			
Troglitazone	Reactive metabolites formation, including quinones and quinone methides, catalysed by CYP3A potentially leading to toxicity	Mainly glucuronidation and sulfation leading to relatively stable metabolites	Inhibition of the BSEP transporter by troglitazone and its troglitazone sulfate Troglitazone sulfate inhibits OATP and is toxic at similar concentrations as the parent compound ₠	Induce apoptotic cell death in human hepatocytes at high concentrations by by ₠! effects on mitochondria resulting in depletion of ATP and release of cytochrome c	Induces mitochondrial membrane permeability in isolated mitochondria ₠	Lipid peroxidation and PPARγ‐dependent steatosis		
Ximelagatran	Stepwise esterase-mediated hydrolysis and *N*-reduction (and vice versa) Outer mitochondrial membrane enzymes—mitochondrial reducing component 1 and 2 (mARC1 and mARC2) involved in melagatran formation	No phase II metabolism reported	Potential involvement of PgP transport metabolism Transport of intermediates thought to be greater than for parent	Cell viability: tolerated well up to at least 200 uM (ATP content) Apoptosis: after exposure of HepG2 cells at 100 uM for 24 h	Toxicity in vitro via mitochondrial mechanism: 1. higher mitochondrial concentrations of melagatran (mARC) 2. asymptomatic hepatic stress (e.g. virus/inflammation) leads to mitochondrial stress Mitochondrial functions: no effects			

### Compounds causing mitochondrial impairment (mechanism 1)

Mitochondrial dysfunction is a general term to include alterations of different metabolic pathways and damage to mitochondrial components (Fig. 3.1). Changes in mitochondrial homoeostasis can have a variety of deleterious consequences, such as oxidative stress, energy depletion, accumulation of triglycerides, and cell death. Mechanisms of mitochondrial dysfunction include membrane permeabilization, oxidative phosphorylation (OXPHOS) impairment, fatty acid β-oxidation (FAO) inhibition and mtDNA depletion. Regarding steatosis, investigations suggest that besides mitochondrial dysfunction several other mechanisms could be involved and they are discussed separately below using the training compounds as umbrella.

**Fig. 3:**
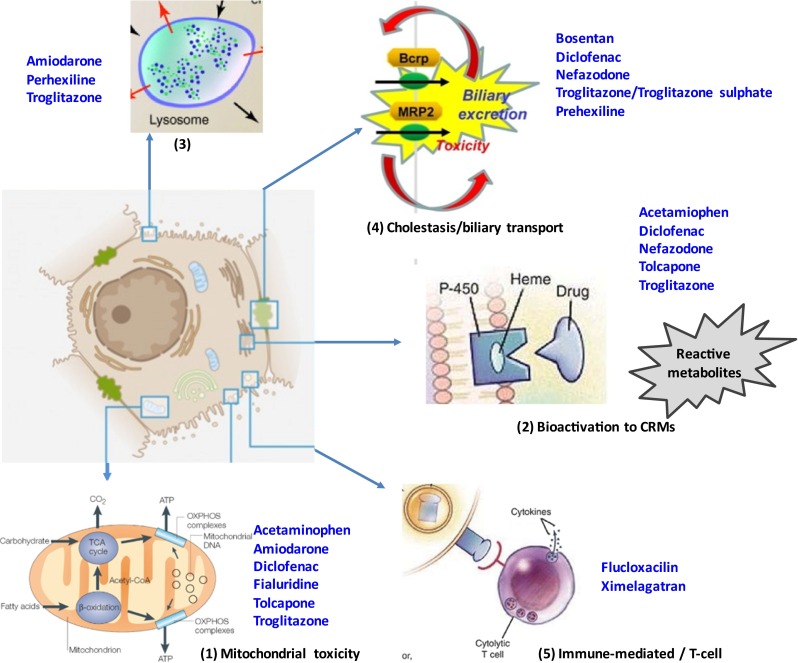
Integrative picture of DILI-related mechanisms and MIP-DILI training compounds

### Fialuridine (mechanism 1)

Fialuridine was developed as an antiviral therapy for hepatitis B infection. In a phase II study, fialuridine caused severe toxicity: irreversible acute hepatic failure in 7 out of 15 patients, myopathy, myoglobinuria, severe lactic acidosis, and neuropathy after 9–13 weeks of treatment (McKenzie et al. 1995). Five out of 7 participants with severe hepatotoxicity died and two survived after liver transplantation liver transplant. Preclinical toxicology studies in mice, rats, dogs, and primates did not provide any indication that FIAU would be hepatotoxic in humans (Trials et al. 1995).

Histologic analysis of human liver tissue showed prominent accumulation of microvesicular fat, with chronic active hepatitis and variable degrees of macrovesicular steatosis, but little hepatocellular necrosis, which is consistent with the absence of substantial elevations in serum aminotransferase levels during treatment.(Kleiner et al. 1997). Electron microscopy showed markedly swollen mitochondria, with loss of cristae, matrix dissolution, and scattered vesicular inclusions.

In studies of fialuridine in a human hepatoma cell line (Hep G2), the drug was incorporated into both nuclear and mitochondrial DNA, but at a much higher rate in the latter (Cui et al. 1995). Morphologic changes in mitochondria, microsteatosis, macrosteatosis, and increased lactic acid production were also observed. The integration of nucleoside analogues into nuclear DNA represents an alternative but potentially delayed pathway to cytotoxicity and cell apoptosis. Expression of a nucleoside transporter hENT1 in human (but not in mouse) mitochondria, which facilitates entry of fialuridine into mitochondria, may be responsible for the human-specific mitochondrial toxicity caused by fialuridine (Lee et al. 2006).

Recently, it has been shown that chimeric mice could be used as a model for fialuridine toxicity. The clinical features, laboratory abnormalities, liver histology, and ultra-structural changes observed were the same as in humans and these abnormalities developed in the regions of the livers that contained human hepatocytes but not in regions that contained mouse hepatocytes (Xu et al. 2014).

### Reactive metabolites (mechanism 2)

Involvement of the liver in drug-related injury rests on the anatomical location of the liver and exposure to orally ingested drugs, physiology and metabolic capacity of the drug-metabolising enzymes. During drug absorption, some of the parent drug is metabolized to typically more hydrophilic entities, the metabolites of which are predominantly inert water-soluble metabolites, but can equally lead to the formation of chemically reactive species, i.e. reactive metabolites (Fig. 3.2). And the process of drug bioactivation to CRMs is believed to be among the number of initiating events of many drug-related liver toxicities. The formation of CRMs can interact with critical intracellular macromolecules leading to toxicity or further interaction with hepatoprotective entities such as glutathione (GSH). CRMs are typically electron deficient molecules (electrophiles) and if not detoxified these electrophiles react with electron rich macromolecules such as proteins, nucleic acids and lipids with the potential to cause a change in biochemical function, or modified such that these modified proteins are processed by the immune antigen presenting cells. CRMs include quinone-imines, quinones, epoxides and reactive oxygen species and other free radicals. Most CRMs are short-lived leading to cellular injury close to the site of formation, but less reactive species can diffuse to other surrounding cells and intracellular organelles depending on concentration, rate of formation of reactive metabolites and the nature of species formed, i.e. hard and soft electrophiles, such as quinones and quinine methides, respectively. Free radicals, by contrast, do not covalently interact with macromolecules, but pair with other free radicals forming covalent bonds with abstraction of hydrogen from neutral molecules to form a new free radical cation.

### Acetaminophen (mechanisms 1, 2, 5)

The association of reactive metabolite formation and APAP toxicity is among the most documented example of drug liver injury (Bessems and Vermeulen 2001). At normal therapeutic doses, APAP is considered safe, but at high doses it is hepatotoxic and accounts for a large proportion of drug-related morbidity in humans (Jaeschke 2015). Registered annual percentage of acetaminophen-related acute liver failure rose from 28 % in 1998 to 51 % in 2003 in multicentre US study (Larson et al. 2005). At therapeutic doses, APAP is conjugated to form the sulphate and glucuronides metabolites and accounts for 40 and 20–40 % of the dose, respectively, in human. GSH conjugation accounts for less than 15 %. Because of cofactor limitation, at high doses of APAP, oxidative metabolism by CYP2E1, 1A2, 2D6, and 3A4 to the cytotoxic *N*-acetyl-p-aminoquinone-imine (NAPQI) becomes more important (James et al. 2003a; McGill and Jaeschke 2013). NAPQI can be reduced by NAD(P)H:quinone oxidoreductase 1 (NQO1) (Powis et al. 1987; Moffit et al. 2007), NAD(P)H:quinone oxidoreductase 2 (NQO2, unpublished) or GSH and be conjugated to GSH resulting in the formation of 3-glutathionyl-paracetamol (APAP-SG) (Coles et al. 1988). Following depletion of GSH, NAPQI reacts increasingly with macromolecules causing subsequent hepatic necrosis. In certain individuals, APAP toxicity can arise with therapeutic doses (Vuppalanchi et al. 2007) and this ‘idiosyncratic’ response in liver injury may likely act as a contributing factor to the differences in the expression and activity of the phase I enzymes.

NAPQI interacts with protein thiols, by covalent binding and thiol-oxidation, including the plasma membrane Ca2^+^-ATPase causing an increase in cytosolic Ca2^+^ concentrations, changes to the actin skeletal structure and function and cell death by centrilobular hepatic necrosis. Covalent binding to critical cellular proteins has been postulated to be the main mechanism of toxicity (Hinson et al. 1995; Pumford and Halmes 1997) in animal species and man. However, the meta-isomer of APAP, *N*-acetyl-meta-aminophenol (AMAP), appears to covalently bind with proteins at levels similar to APAP, but without toxicity in mice or hamster, yet still it forms the analogous reactive metabolite in rat and human precision-cut liver slices. It appears that AMAP covalently binds and inactivates CYP2E1 in mice, which contrasts the macromolecular covalent binding associated with NAPQI and ensuing necrosis located in the centrilobular region of the liver (Salminen et al. 1998; Hadi et al. 2013). While covalent binding has been attributed to the toxicity of NAPQI, mitochondrial dysfunction as a consequence of APAP toxicity suggests a role of NAQPI-targeted mitochondria through modification of proteins associated with the electron transport chain, namely complex V, in addition to nitrated residues on Complex 1 in response to oxidative stress in mice (Qiu et al. 2001; Liu et al. 2009). More recently, APAP was shown to up-regulate the electron transport chain protein expression, possibly in response to oxidative stress and presence of unstable adducts with cysteinyl thiol groups (Stamper et al. 2011).

Studies also suggested a role of the innate immune system in APAP toxicity (Jaeschke et al. 2012). Pro- and anti-inflammatory cascades are simultaneously activated, and their balance plays a major role in determining the progression and severity of APAP-induced hepatotoxicity. A number of modulators of inflammatory responses have been described that can alter the severity of liver injury following the initiation of toxicity. Up-regulation of TNFα and IL-1α occurs in the acetaminophen-treated mouse. However, the role of TNFα in APAP toxicity is somewhat controversial (Boess et al. 1998; Simpson et al. 2000). Other pro-inflammatory cytokines: interleukin one beta (IL-1β) and interferon gamma (IFNγ) have also been examined in APAP toxicity (Blazka et al. 1995; Ishida et al. 2002; James et al. 2003b; Gardner et al. 2003). It was shown that depletion of interleukin (IL-6) resulted in increased sensitivity to APAP (Masubuchi et al. 2003). Chemokines, e.g. macrophage inhibitor protein 2 (MIP-2), also play a role in acetaminophen-induced toxicity and are up-regulated in APAP toxicity (Lawson et al. 2000).

Intracellular signalling mechanisms also play a role in APAP toxicity: the c-Jun *N*-terminal kinases (JNKs), a subfamily of the mitogen-activated protein (MAP) kinases, are activated by phosphorylation early in APAP toxicity (Gunawan et al. 2006; Latchoumycandane et al. 2006; Henderson et al. 2007) and DNA fragmentation is another mechanism that has been implicated in acetaminophen-induced hepatotoxicity (Salas and Corcoran 1997). Subsequently, it was reported that endonuclease G was important in the acetaminophen-induced nuclear fragmentation (Bajt et al. 2006).

As described, the hepatotoxicity of APAP appears to occur by a complex mechanistic sequence. Associated with these essential events, there appears to be a number of modulators of inflammatory responses that can alter the severity of liver injury. The threshold and susceptibility to APAP hepatotoxicity is determined by the interplay of injury promoting and inhibiting events downstream of the initial production of toxic metabolite where environmental and genetic control may be of critical importance in determining susceptibility to APAP hepatotoxicity.

### Diclofenac (mechanisms 1, 2, 4)

Therapeutic use of diclofenac (DF) is associated with rare but sometimes fatal hepatotoxicity with a characteristic delayed onset of symptoms and poor dose–response relationship. Elevated levels of liver enzymes develop in about 15 % of patients that are regularly taking diclofenac and a threefold rise in transaminase levels has been reported in 5 % (Banks et al. 1995). Clinically relevant hepatotoxicity leading to hospital referral occurs in 6.3 per 100,000 diclofenac users (de Abajo et al. 2004).

In contrast to APAP, diclofenac requires initial hydroxylation by CYP2C9 and CYP3A4, or peroxidase-mediated oxidation to form different quinone-imine reactive metabolites from 4′ and 5-hydroxydiclofenac, which in turn can form adducts with macromolecular proteins or form conjugates with GSH (Tang et al. 1999; Madsen et al. 2008b). The parent diclofenac also forms a reactive acyl-glucuronide metabolite with the formation of covalent modification of cellular proteins and the covalent binding to liver proteins in rats, which is linked to the activity of a hepatic canalicular transport protein, Mrp2 (Tang 2003). The acyl-glucuronide and the quinone-imines of diclofenac, derived from metabolic activation of diclofenac, are both implicated in covalent modification of cellular proteins with the disruption of critical cellular functions and/or immunological response in susceptible patients (Shen et al. 1999; Kenny et al. 2004). Besides GSH conjugation, it has been shown that quinone-imines of 4′ and 5-hydroxydiclofenac can be detoxified by reduction by polymorphic NQO1 as well (Vredenburg et al. 2014).

Besides distinct chemically reactive metabolites, other diclofenac-related hazards have been identified and have been studied. These include oxidative stress generation based on peroxidase-catalysed production of radicals, which in turn can oxidize GSH and NAD(P)H, while molecular oxygen may be reduced and activated or the radicals undergo redox cycling (Galati et al. 2002). Also, mitochondria are a major subcellular target for diclofenac. Diclofenac disturbs in vitro liver mitochondrial function at multiple levels, including the phosphorylating system and mitochondrial permeability transition pore, being associated with increased oxidative stress and apoptotic signalling (Masubuchi et al. 2002; Gómez-Lechón et al. 2003). In high concentrations, diclofenac causes rapid and concentration-dependent ATP depletion. Diclofenac decreases the mitochondrial transmembrane potential by direct effects on the mitochondrial inner membrane, uncoupling of respiration by proton shuttling or opening of the mitochondrial membrane permeability transition pore. In HepG2 cells, TNFα enhances hepatocyte injury caused by diclofenac (Fredriksson et al. 2011). Diclofenac-mediated stress signalling suppressed TNFα-induced survival signalling routes and sensitizes cells to apoptosis. However, a striking but somewhat sobering conclusion is that we still do not understand the real reasons for the individual susceptibility in patients.

### Lysosomal impairment (steatosis and phospholipidosis; mechanism 3)

Microvesicular steatosis or microsteatosis is a form of liver toxicity which is associated with liver failure, pronounced hypoglycaemia and encephalopathy in patients (Farrell 2002; Stravitz and Sanyal 2003). Liver pathology reveals the presence of numerous cytoplasmic lipid droplets, visible by staining with oil red O. Liver triglycerides can accumulate as vacuolar lipid bodies within the hepatocyte by a number of drugs (Farrell 2002; Labbe et al. 2008) and frequently referred to as non-alcoholic fatty liver (NASH), which is most frequently observed in obese, diabetic patients and related disorders. Progression from fibrosis to cirrhosis can occur rapidly. Drugs responsible for this hepatic lesion can also induce a mixed form of fat accumulation with macrovacuolar steatosis and microvesicular steatosis occurring in anatomically adjacent hepatocytes, the formation of which may depend on proteins such as perilipin and adipophilin associated with lipids and possible presence of free fatty acids (Fromenty and Pessayre 1995). The mechanisms associated with macrovacuolar and microvesicular steatosis remain to be confirmed, but steatosis arises from either an increased availability of free fatty acids to the liver and stimulation of *de novo* hepatic lipogenesis (Begriche et al. 2006; Moreau et al. 2009). Drugs known to cause steatosis can be broadly divided into those with steatosis and steatohepatitis with well-characterized mechanisms of hepatotoxicity, for example, amiodarone, fialuridine and perhexiline. Other drugs form less well-defined mechanisms include latent forms of NASH, such as tamoxifen, and episodic cases of steatosis and steatohepatosis, such as carbamazepine.

Cationic amphiphilic compounds comprise a lipophilic moiety and amine group, the latter of which becomes protonated as the drug crosses from the cytosol into the acidic milieu of lysosomes or crosses the outer membrane of the mitochondrion into the acidic inter-membrane space where the uncharged drug is subsequently protonated and unable to pass back across the membrane as the positively charge species. As a consequence of the charge distribution, drug accumulates in lysosomes as the positively charged drug forming noncovalent complexes with phospholipids (Fig. 3.3). As the drug-phospholipid complexes are not degraded, these complexes progressively accumulate in the form of myelin structures of enlarged lysosomal bodies. The clinical pathology of cationic amphiphilic accumulation of drugs, as phospholipids, leads to the common occurrence of phospholipidosis in patients, yet the clinical consequences of which appears with limited clinical symptoms. By contrast, cationic amphiphilic drugs crossing the outer mitochondrial membrane are protonated and through the electrochemical gradient pass to the inner mitochondrial matrix where these drugs accumulate targeting processes of mitochondrial function linked with e-transport chain and fatty acid metabolism.

### Amiodarone (mechanisms 1, 3)

Amiodarone is a cationic amphiphilic lipophilic compound (Atiq et al. 2009) with the propensity to accumulate in the lipid-rich environment of organelles affecting both mitochondrial and lysosomal function and thus causes liver damage by a number of mechanisms: microvesicular steatosis, concomitant macrovacuolar steatosis and steatohepatitis, and phospholipidosis. Elevated blood levels of liver enzymes have been recorded in 14–82 % of patients (Lewis et al. 1989).

As a consequence of the charge distribution from cytosol to the acidic milieu, amiodarone accumulates and inhibits phospholipase activity through one of two mechanisms: firstly, by formation of noncovalent complexes with phospholipids in lysosomes and as the drug-phospholipid complexes are not degraded, these complexes progressively accumulate as described above (Pirovino et al. 1988), leading to phospholipidosis; secondly, amiodarone and its metabolites (e.g. *N*-desmethylamiodarone) accumulate in lysosomes of parenchymal, bile duct epithelium and kupffer cells with inhibition of the metabolism of lysosomal lipids by phospholipases A1 and A2 leading to phospholipidosis (Heath et al. 1985).

Phospholipidosis can occur within 2 months of starting amiodarone therapy (Capron-Chivrac et al. 1985; Rigas et al. 1986) and occurs in a higher percentage of patients receiving amiodarone than the incidence of hepatocellular damage (Atiq et al. 2009), suggesting amiodarone-induced phospholipidosis may only have a contributory role towards the more serious consequences of amiodarone-induced hepatotoxicity and cirrhosis. Therefore, diagnosis of phospholipidosis in patients serves as a biomarker for the accumulation of amiodarone (Atiq et al. 2009) rather than as a biomarker for the more serious forms of amiodarone hepatotoxicity.

With formation of enlarged lysosomal bodies, the release of proteolytic enzymes from aberrant lysosomes is a mechanism likely attributed to amiodarone-induced liver damage (Guigui et al. 1988; Yagupsky et al. 1985; Lewis et al. 1990). With the seepage of proteolytic enzymes over prolonged periods, the proteolytic enzymes may in turn contribute to the elevation of aminotransferases leading to hepatic necrosis, fibrosis, and cirrhosis of the liver.

Amiodarone-induced inhibition of cellular respiration is another possible pathogenic mechanism for amiodarone-induced liver damage. Impairment of mitochondrial β-oxidation and uncoupling of oxidative phosphorylation leads to the formation of reactive oxygen species, which in turn has a role in the development of amiodarone-induced cirrhosis (Fromenty et al. 1990a, b).

In addition to the usual pathologic findings of cirrhosis, leucocytic infiltrate and high Mallory’s hyaline (Mallory’s bodies) are suggestive of amiodarone-induced cirrhosis together with the presence of phospholipid-rich lamellar lysosomal inclusion bodies (Lewis et al. 1990).

Although amiodarone hepatotoxicity is serious and potentially fatal, such effects are rare. Asymptomatic liver enzyme elevation occurs in 25 % of the population treated with amiodarone (Lewis et al. 1989) and is usually reversible upon discontinuation of therapy (Pollak 2010). Symptomatic hepatic dysfunction occurs in less than 1 % of the population treated with amiodarone. Besides chronic liver injury which includes steatosis (macro and microvesicular steatosis) and cirrhosis due to prolonged amiodarone use, acute hepatic side effects (idiosyncratic reaction may be involved in pathogenesis) from amiodarone intravenous loading dose have been reported (Rätz Bravo et al. 2005).

### Perhexiline (mechanisms 3, 4)

Perhexiline is an anti-anginal drug, which despite its efficacy diminished in its use due to a small number of cases of severe hepatotoxicity and neurotoxicity. Perhexiline is mainly metabolized by the polymorphic CYP2D6 in the liver, and these side effects were related to high plasma concentrations with standard doses in poorly metabolizing patients (Wright et al. 1973; Shah et al. 1982).

At high concentrations, protonated perhexiline rapidly accumulates in mitochondria along the mitochondrial membrane potential as an amphiphilic (or amphipathic) molecule. A multitude of effects on mitochondria have been reported, including the uncoupling of mitochondrial oxidative phosphorylation, inhibition of complexes I and II, and decreased ATP formation. The most evident effect was fatty acid metabolism inhibition (Ashrafian et al. 2007). Most importantly, characteristic lamellar lysosomal inclusion bodies representing phospholipidosis as well as triglyceride and fatty acid accumulation were identified.

### Cholestasis, inhibition of biliary efflux and BSEP (mechanism 4)

Cholestatic and mixed cholestatic and hepatocellular injury are forms of severe DILI in man. Recent evidence of drugs secreted into the bile suggests these drugs are primary candidates inducing cholestatic liver disease in patients, but do not induce hepatotoxicity in rats. The flow of bile is highly regulated through several basolateral and canalicular transport proteins such as Na^+^-dependent taurocholate transport protein, BSEP and a series of multi-drug resistance associated proteins. Among these transport proteins, BSEP is believed to play a pivotal role for DILI and inhibition of this transport protein leading to cholestatic injury. Bile acid accumulation in hepatocytes as a consequence of BSEP inhibition is proposed as a mechanism for the hepatotoxicity of several drugs including cyclosporine, troglitazone and bosentan (Fig. 3.4). Of those drugs causing cholestasis in patients, several do not cause a similar pattern of cholestatic injury in the rat, but nevertheless inhibit Bsep with reported elevations in the levels of serum bile acids. Explanations for the lack of toxicity in the rat stem from either the inhibition of uptake transport proteins and/or differences in the inhibitory potential of human BSEP and rat Bsep. Alternative explanations for the lack of hepatotoxicity in the rat are provided by the complement of bile acids comprising the bile acid pool in the liver—rat bile acid composition being more hydrophilic and therefore less toxic than the composition of human bile acids. Nevertheless, the overall interplay and mechanisms of drug-induced cholestasis remain poorly defined. Among several studies on the role of BSEP hepatocellular injury, two substantive studies link hepatotoxicity to BSEP inhibition. Of those drugs evaluated, 75 % of drugs exhibit IC50 greater than 133 µM and therefore were viewed negative. By contrast, 16 % showed greater inhibition of BSEP with IC50 ≤25 µM (Morgan et al. 2010) and in a further study, 17 of 85 drugs with an IC50 <100 µM and *C*_max,u_ >2 nM are known to cause DILI in man (Dawson et al. 2012). Both these studies, and others, conclude that BSEP inhibition plays a role in cholestatic injury in patients.

### Bosentan (mechanism 4)

The proposed mechanism of bosentan-induced cholestasis is presently thought to be mediated, at least in part, by inhibition of BSEP activity and is among one of the more extensively investigated drugs to inhibit BSEP. The mechanism of bosentan-induced liver injury in patients is believed, at least in part, to be mediated inhibition of the canalicular bile transport protein BSEP with accumulation of bile acids as the prevailing mechanism in the aetiology of bosentan-induced liver injury in patients (Fattinger 2001). Consistent with this, proposed mechanism is the observed inhibition of BSEP in vitro models and the incidence of dose-related liver injury in patients, which is reversible with reduction of daily dose (Noé et al. 2002; Mano et al. 2007). The inhibition of BSEP is further supported by clinical observations demonstrating the increase in serum bile acids and the toxicity of bosentan is more commonly observed in patients co-treated with glyburide, a known inhibitor of BSEP (Stieger et al. 2000). Bosentan is mainly eliminated by hepatic metabolism and subsequent biliary excretions of three metabolites formed by CYP 2C9 and 3A4 (Pichler et al. 1988) with no evidence of reactive metabolite formation.

The intravenous administration of bosentan to rats increases the level of serum bile acids suggesting the rat as a possible model for the study of BSEP inhibition and hepatotoxicity in man (Fattinger 2001). However, treatment of rats with bosentan does not cause liver injury where a basolateral compensatory role of Ntcp, expressed more than human NTCP, contributes to the sinusoidal transport of bile acid elimination and a reduction in intracellular accumulation of bile acids in the liver of rats (Hagenbuch and Meier 1994). In the rat, bosentan is a more potent inhibitor of Ntcp than the human NTCP in suspensions of hepatocytes and in vesicles expressing Ntcp and NTCP proteins with mechanisms of non-competitive and competitive inhibition, respectively (Leslie et al. 2007).

Mild forms of bosentan-associated liver toxicity occurs in approximately 10 % in patients within 6 months, but less frequently serious liver toxicities are reported and are often associated with co-morbidities (Eriksson et al. 2011). The idiosyncratic nature of bosentan; delay of onset, role of comorbidities and variable extent of the mild to severe forms of liver toxicities and perceived mechanism of bosentan-induced liver toxicity was selected as the a priori training compound for inclusion for the study of BSEP and drug transport studies.

Nefazodone, troglitazone, diclofenac, tolcapone and perhexiline are among a number of other drugs selected as training compounds with implications for the inhibition of bile transport proteins.

### Tolcapone (mechanisms 2, 4)

Tolcapone is a selective and reversible inhibitor of the enzyme catechol-*O*-methyl transferase (COMT) and is used as an effective adjunct to levodopa/carbidopa in the treatment of Parkinson’s disease. However, tolcapone is under strict regulations on liver enzyme monitoring due to the hepatic failures that appeared, three of them with fatal outcome. In 1998, tolcapone was actually withdrawn from the European Union (EU) and Canadian markets due to liver problems, but it is again reintroduced to EU. Liver function elevations above the upper limit of normal (ULN) occurred in 20.2 and 27.5 % of patients in the placebo and active treatment groups, respectively; increases ≥3 times the ULN occurred in 1.2 and 1.7 % of patients (Lees et al. 2007).

The mechanism of hepatotoxicity introduced by tolcapone is still not well understood, but it seems that tolcapone is able to cause mitochondrial uncoupling of OXPOS and to disrupt the energy-producing cycle (Haasio et al. 2002a, b). This leads to a decreased ATP production and increased oxygen consumption as a compensatory function of the cell, and ultimately cell death may take place. Uncoupling of OXPOS is reflected as a rise in body temperature since the energy is released as heat and liver damage is induced due to mitochondrial toxicity (Terada 1990).

Tolcapone has been reported to be toxic to human neuroblastoma cells and caused a profound reduction in ATP synthesis mirroring the effects of a classical uncoupler (Korlipara et al. 2004). However, the same study showed that tolcapone markedly inhibits ATP synthesis in cultured cells devoid of mtDNA and therefore, a functional respiratory chain. Tolcapone-induced hepatotoxicity could also be related to elevated catecholamine levels in patients which receive other drugs with adrenergic receptor-mediated toxicity (Rojo et al. 2001). The mechanism of tolcapone toxicity may thus also involve mechanisms independent of its effects on OXPHOS.

It has also been speculated that the different metabolism of second-generation COMT inhibitors might be responsible for the toxicity observed. Metabolism of tolcapone into amine or acetylamine metabolites in humans can be followed by oxidation to reactive oxygen species and induce hepatocellular injury. The same oxidative metabolites are not found in humans treated with entacapone (Smith et al. 2003). Also, mutations in the uridine diphosphate-glucuronosyltransferase 1A9 gene which encodes for the enzyme which metabolizes tolcapone and thereby might promote enhanced COMT activity showed increased occurrence of hepatic dysfunction (Martignoni et al. 2005). Besides the effect on OXYPHOS, tolcapone is reportedly an inhibitor of BSEP transporter with comparatively higher value of IC50 than several more potent inhibitors of BSEP activity in vesicle systems (Morgan et al. 2010).

### Nefazodone (mechanisms 2, 4)

Nefazodone is a triazolopyridine antidepressant withdrawn from the market due to the significant number of reports of nefazodone-mediated hepatic injury (Stewart 2002; Choi 2003). Described clinical symptoms were jaundice and increases in ALT (10×), AST (10×), total bilirubin (mostly conjugated), and prothrombin time. Histological liver evaluations demonstrated centrilobular necrosis, bile-duct proliferation with cholestasis (Lucena et al. 1999). The incidence was reported 1 in 250,000–300,000 patients-years of exposure and the onset of injury varied from 6 weeks to 8 months. Although the exact mechanism of hepatotoxicity remains unknown, several possible mechanisms have been described in the literature.

It has been shown that clinical hepatotoxicity of nefazodone is linked to the ability of drug to inhibit bile acid transport (Kostrubsky et al. 2006). Nefazodone induces a strong inhibition of BSEP and taurocholate efflux in human hepatocytes, and 1 h after oral drug administration transitory increase in rat serum bile acids was observed (Kostrubsky et al. 2006).

Mitochondrial impairment is likely to contribute to nefazodone hepatotoxicity (Dykens et al. 2008). In isolated rat liver mitochondria and in intact HepG2 cells, nefazodone inhibits mitochondrial respiration. Using immunocaptured oxidative phosphorylation complexes, complex I, and to a lesser amount complex IV were identified as the targets of toxicity associated with accelerated glycolysis. Bioactivation of nefazodone and formation of reactive intermediates have also been described. Nefazodone incubations with microsomes or recombinant CYP3A4 in the presence of sulphydryl nucleophiles showed formation of thiol conjugates of mono-hydroxylated nefazodone metabolite (Kalgutkar et al. 2005).

### Troglitazone (mechanisms 1, 2, 3, 4)

Troglitazone was the first thiazolidinedione anti-diabetic drug that was removed from the market due to reported cases of increase in ALT and 1 in 40, 000 patients with reported liver failure (Faich and Moseley 2001). In clinical trials, 1.9 % of the subjects had elevations of ≥3× ULN of ALT concentrations and reported cases of overt liver injury and jaundice (Watkins 2005).

Formation of reactive intermediates, including quinones and quinone methides, is hypothesized to be responsible for troglitazone hepatotoxicity through either GSH depletion/covalent binding mechanism or via oxidative stress caused by redox cycling of the quinone. However, there was no correlation of the generation of the reactive metabolites with susceptibility to troglitazone cytotoxicity, and chemical inhibitors of drug metabolizing enzymes in in vitro could not protect the cells against the toxicity (Kostrubsky et al. 2000; Tettey et al. 2001). Therefore, metabolic activation of troglitazone is not apparently the primary mechanism of hepatotoxicity. Several other mechanisms have been described.

Troglitazone induces cytotoxicity in hepatocytes from numerous species including humans. A major non-metabolic toxicity factor is via effects on mitochondria resulting in depletion of ATP and release of cytochrome c, which induces cell death via apoptosis (Tirmenstein et al. 2002; Hu et al. 2015). Lipid peroxidation and PPARγ-dependent steatosis are also mediated by troglitazone. Troglitazone induced PPARγ levels selectively in the liver under pathophysiological conditions, and severe steatosis may result in the accumulation of the drug in lipid-rich hepatocytes, with subsequent lipid peroxidation, and predispose the liver to the development of fibrosis (Bedoucha et al. 2001; Boelsterli and Bedoucha 2002). Susceptibility to liver injury in individuals has been attributed to explain the distinct sensitivity of patients to troglitazone. It has been shown that diabetics, obese individuals, and other persons with impaired liver function, were more likely predisposed to troglitazone toxicity due to decreased ability to eliminate the drug, compromised mitochondrial function in the liver cells, bile salt retention and steatosis, and/or underlying inflammatory status of the liver in diabetic subjects.

Other mechanisms linked to mechanisms of hepatotoxicity are the inhibition of the BSEP transporter by troglitazone and its metabolite, troglitazone sulphate, with the accumulation of toxic bile salts in the liver cells, cholestasis and apoptosis through the Fas death receptor signalling pathway (Funk et al. 2001; Yang et al. 2014). Inhibition of BA transport by troglitazone and its major metabolite, troglitazone sulphate, has recently been shown through use of Quantitative Systems Pharmacology (QSP) to predict delayed hepatotoxicity in humans due to hepatocellular accumulation of toxic bile acids and drug exposure, and species differences attributable to the pathophysiology of bile acids (Yang et al. 2014).

### Adaptive immunity (mechanism 5)

It is clear from the literature that immune responses and associated autoimmunity play an important role in both predictive (acute) and idiosyncratic DILI (Fig. 3.5). There is an increased weight of evidence for the role of immune cells (lymphocytes, macrophages, and neutrophils) in hepatic pathology.

Although hepatic inflammation is a common finding in drug-induced liver toxicity, the classic immune-allergic or hypersensitivity reactions are generally found in only a minority of DILI patients. The inflammatory phenotype has been attributed to the innate immune response generated by Kupffer cells, monocytes, neutrophils, and lymphocytes. The adaptive immune system is also influenced by the innate immune response leading to liver damage. Drugs that cause idiosyncratic DILI associated with fever, rash, a relatively short period of therapy before the onset of DILI and a rapid onset on re-challenge fit into the immune idiosyncrasy category.

### Ximelagatran (mechanism 5)

Ximelagatran was the first oral direct thrombin inhibitor that reached the market for short-term use in the prevention of venous thromboembolism and was assessed for the prevention and treatment of a range of thromboembolic disorders for chronic use. Pre-clinical and toxicological studies provided no indication of ximelagatran affecting hepatic functions. Also, in short-term prophylaxis, there was no increase in the incidence of liver enzyme elevations with ximelagatran. However, clinical trials with long-term (>35 days) treatment with ximelagatran showed increased rates of liver enzyme elevations (Wallentin et al. 2003; Schulman et al. 2003; Olsson 2003; Lee et al. 2005; Albers et al. 2005; Fiessinger et al. 2005). The clinical pattern of events suggests hepatocellular damage. The combination of ALT >3 ULN and total bilirubin >2 ULN was 0.5 % among patients treated with ximelagatran (Keisu and Andersson 2010). Return of ALT to normal was documented in the majority of patients whether treatment was maintained or discontinued, suggesting an adaptive mechanism. Extensive in vitro studies at the molecular, subcellular and cellular level have not been able to define mechanisms explaining the pattern of hepatic injury observed in these long-term clinical trials (Kenne et al. 2008).

Previously reported mechanisms of drug-induced hepatotoxicity are unlikely to explain the observed ALT elevations in ximelagatran exposed individuals: ximelagatran metabolism does not involve the CYP450 system and does not form reactive metabolites, no effects have been observed below 100 μM ximelagatran when investigating cell viability, mitochondrial function, calcium homoeostasis, apoptosis, cytoskeleton, reactive oxygen species, GSH levels, bile acid transporters and nuclear receptors. The knock down of mARC2, the enzyme that reduces ximelagatran, has recently illustrated that the mitochondrial toxicity is strongly inhibited, suggesting a component of metabolic activation and decrease in GSH levels (Neve et al. 2015).

A possible immunogenic pathogenesis, a strong genetic association between elevated ALT and the major histocompatibility complex (MHC) alleles DRB1*07 and DQA1*02 was discovered and replicated, suggested an underlying immune-related mechanism but with no clinical signs of immunopathology (Kindmark et al. 2008).

Dabigatran etexilate is another novel direct thrombin inhibitor DTI proven to be effective and liver-friendly in various randomized controlled clinical trials mainly in the settings of venous thromboembolism and atrial fibrillation (Ma et al. 2011). Although not formally on the list of negative controls, it is used in some of the MIP-DILI experiments as a comparison for ximelagatran.

### Flucloxacillin (mechanism 5)

Flucloxacillin is a β-lactam antibiotic, used as the first line treatment for Staphylococcal infections. Common use of drug induced cholestatic liver injury in 8.5 in 100,000 patients with the delayed onset of clinical symptoms 1–45 days and 1.8 following 46–90 days after starting flucloxacillin therapy (Koek et al. 1994; Dobson et al. 2005; Russmann et al. 2005; Li et al. 2009). The mechanism or mechanisms of cholestasis with bile duct injury and Vanishing Bile Duct Syndrome (ductopenia) are largely unknown, yet small amounts of the compound form metabolites that involved the activity of CYP3A4, which itself may be under genetic control. Whether the formation of these metabolites is directly toxic to cholangiocytes after excretion into bile or formed in cholangiocytes is not yet known. It is generally accepted that an immune-mediated response subsequently accounts for the development of the directly or indirectly linked genetic pre-disposition of DILI, but the mechanisms are still to be conclusively determined.

Strong HLA association with DILI and activation of CD8+ T cells isolated from patients with DILI are suggestive for adaptive immune activation. HLAB*57:01 genotype carriers have an approximately 80-times higher risk (odd ratios = 80.6) of flucloxacillin-induced DILI, but not all carriers will develop DILI (Daly et al. 2009). Evidence showed that reactions to flucloxacillin are driven by drug-specific activation of CD8+ T lymphocytes. Flucloxacillin-responsive CD4+ and CD8+ T cells from patients with DILI have been characterized and shown that naive CD8+ T cells from volunteers expressing HLA-B*57:01 are activated with flucloxacillin (Monshi et al. 2013). Covalent modification of flucloxacillin is thought to be a prerequisite for flucloxacillin-induced liver injury (Carey and Van Pelt 2005). Flucloxacillin modifies specific lysine residues on human serum albumin (Monshi et al. 2013). However, relationship between adduct formation in liver, immune activation and liver injury not defined yet.

Flucloxacillin DILI is the only model of idiosyncratic immune-mediated DILI with patient data to confirm an immune pathogenesis.

### Negative control compounds

Buspirone, entacapone, metformin and pioglitazone were proposed as negative controls. Some are structural analogues of training compounds with less or no toxic effects at the therapeutic dose when compared to the matched positive training compound. Others are not structural analogues, but do address the same pharmaceutical target without evidence of known liver toxicity.

It is important to mention that probably no compound is a ‘true negative’ with regard to cellular toxicity and dose. Equally, no compound will necessarily target exclusively one pathway, so there will always be ‘biological background’ or multiple toxicological events, and the compensatory mechanisms invoked.

### Buspirone

Buspirone, the azaspirodecanedione anxiolytic and antidepressant, is a 5-HT1A receptor partial agonist and a mixed agonist/antagonist on postsynaptic dopamine receptors. Without any reports or clinical observations associated with DILI, it is commonly utilized as negative control drugs (Wu et al. 2016).

Buspirone is a marketed structural analogue of nefazodone, and it is commonly used as its pair in the studies, together with trazodone. Inhibition of canicular transport with nefazodone has been reported, but not with trazodone or buspirone (Kostrubsky et al. 2006). Accordingly, nefazodone was the most toxic, trazodone had relatively modest effects, whereas buspirone showed the least cytotoxicity and effects on mitochondrial function (Dykens et al. 2008).

Although buspirone, like nefazodone, generates p-hydroxybuspirone in liver microsomes, no sulphydryl conjugates of this metabolite were observed suggesting that two-electron oxidation of p-hydroxybuspirone to the corresponding quinone-imine is less favourable. It was also observed that the 2-aminopyridine or 2-aminopyrimidine derivatives present a ‘safer’ alternative to aniline-based compounds, which are prone to bioactivation (Kalgutkar et al. 2005), and perhaps confirms the lack of idiosyncratic hepatotoxicity with buspirone in the clinic.

### Entacapone

Entacapone is a selective, potent and reversible COMT inhibitor and structural analogue of tolcapone. While tolcapone is under strict regulations on liver enzyme monitoring, due to the reported cases of hepatotoxicity, entacapone has not been related to reported cases of associated liver injuries in patients. For this reason, entacapone was selected as a negative training compounds used in the MIP-DILI project.

In clinical use, entacapone has only been reported to induce hepatotoxicity in 3 cases (Fisher et al. 2002). However, in two of these cases, the patients had concomitant medications with hepatotoxic potential, and the third case was reported with a history of long-standing alcohol abuse and alcohol-induced liver cirrhosis.

In vitro assays have shown that entacapone is a weak uncoupler of oxidative phosphorylation at high concentrations, while tolcapone has been reported to be an uncoupler at low micro-molar concentrations (Nissinen et al. 1997; Haasio et al. 2002a). Sets of proteins interacting with entacapone or tolcapone were identified in human cell line HepG2 and rat liver subcellular fractions. The cellular distribution of proteins captured by entacapone was not linked to mitochondrial function, while for tolcapone, a large proportion of proteins were identified to be mitochondrial origin (Fischer et al. 2010). Also, in in vivo studies in rats tolcapone were more toxic than entacapone causing high mortality, elevation of body temperature and necrotic changes in liver tissue (Haasio et al. 2001, 2012b).

Difference in toxicity could also be a result of differences in the metabolism of drugs. Amine or acetylamine metabolites that can be followed by oxidation to reactive oxygen species and induce hepatocellular injury by being trapped by GSH to form metabolic adducts were not observed in with entacapone (Smith et al. 2003). Entacapone clearance is also significantly higher than tolcapone clearance in humans (Data from FDA approved labelling). In addition, recent work has demonstrated that both drugs have the potential to alter hepatobiliary transport causing modest inhibition of the BSEP and the basolateral efflux transporters (MRP3 and MRP4) (Morgan et al. 2013).

### Metformin

Metformin is an antihyperglycaemic agent, which improves glucose tolerance in patients with type 2 diabetes, lowering both basal and postprandial plasma glucose. Because metformin is not metabolized in the liver (Sirtori et al. 1978), it has been considered safe from a hepatic liver injury although known cases of cholestasis have been reported (Babich et al. 1998; Desilets et al. 2001; Nammour et al. 2003; Kutoh 2005).

Metformin can promote liver mitochondria injury and predispose to cell death (Carvalho et al. 2008; Bridges et al. 2014). Biguanide-induced mitochondrial dysfunction yields increased lactate production and cytotoxicity of aerobically poised HepG2 cells and human hepatocytes in vitro (Dykens et al. 2008). However, human vivo and clinical side effects showed fewer than 10 cases of metformin-induced hepatotoxicity (mixed pattern) (Saadi et al. 2013). These cases are idiosyncratic, usually associated with alcohol or other drugs, and mainly occur in older patients (Kutoh 2005; Cone et al. 2010; Saadi et al. 2013).

### Pioglitazone

Pioglitazone, a second-generation thiazolidinedione, is commonly used in the management of type 2 diabetes mellitus. Unlike troglitazone, pioglitazone is generally considered safe from a hepatic standpoint and is commonly used as negative compound for liver toxicity studies. Although case reports of liver injury and failure with pioglitazone have been published (Maeda 2001; May et al. 2002; Nagasaka et al. 2002; Pinto and Cummings 2002; Floyd et al. 2009), the risk of liver failure or hepatitis is not higher than with other oral antidiabetic agents (Rajagopalan et al. 2005; Berthet et al. 2011).

In vitro studies in hepatocyte cultures showed that troglitazone alone among the thiazolidinediones is toxic (Kostrubsky et al. 2000). The difference in the profiles is the presence of the side chain of troglitazone, which might uniquely predispose it among the thiazolidinediones to hepatotoxicity due to the quinone metabolite formation. From electrochemical oxidations of pioglitazone in the presence of GSH, no GSH conjugates could be identified (Madsen et al. 2008a).

Moreover, the observed ALT elevation levels in troglitazone clinical trials, results of studies in hepatocyte cultures and evidence of the distinct metabolic pathway suggest that hepatotoxicity may not be a class effect of the thiazolidinediones but rather a unique effect of troglitazone and that pioglitazone do not share its hepatotoxic profile (Scheen 2001; Tolman and Chandramouli 2003).

## Summary and conclusions

Training compounds were selected on the basis of clinical evidence of DILI and availability of known mechanisms by which these compounds are believed to cause liver injury. The evidence-based selection of the set of training compounds was subsequently employed systematically for the pharmacological, physiological and toxicological evaluation of model systems to support the translational relevance of these models and improvement of a greater understanding of drug liability’s mechanisms to cause DILI in man. From a total of 40 compounds nominated, 10 training compounds and 4 negative controls were selected.

It is acknowledged at the outset in the selection of drug compounds that our present knowledge of the mechanisms by which drugs are known to cause human DILI remains incomplete. Moreover, through the evidence-based selection of training compounds, many can be regarded as largely imperfect ‘reagents’ to probe, in isolation and selectively, each of the 5 categories of DILI, where many of the drugs reviewed herein neither exhibit a specific biochemical or toxicological target. As such, many if not all drugs in this review appear to exhibit multiple toxicological mechanisms when evaluated in model systems or clinical intelligence (see Fig. 3). Thus it is clear that prediction of DILI in man requires a matrix of multiple test systems and training compounds to afford a deeper understanding of which mechanisms/liabilities are relevant to man. The ‘selection’ of training compounds therefore offers a valuable tool to profile DILI mechanisms and to interrogate existing and novel in vitro systems for the prediction of human DILI. This a priori knowledge of drugs to target more than one mechanism leading to probable DILI also underlies the basis for a more rational integrated approach to the investigation of DILI early in drug discovery to eliminate simple and more complex liabilities through the use of a tiered approach to early safely risk assessment.

Several publications have recently appeared in which the here selected training compounds and negative controls have been investigated in order to substantiate their utility as training compounds to profile and elucidate further DILI mechanisms and to interrogate existing and novel in vitro systems for the prediction of human DILI (Sison-Young et al. 2015; Bell et al. 2016; Sison-Young et al. 2016; den Braver-Sewradj et al. 2016; den Braver et al. 2016).
